# 

**Published:** 1882-04

**Authors:** 


### Pamphlets.

Croupous Pneumonia: Is it a zymotic disease? By D. W. Prentiss, A.M., M.D. Reprint from Transactions of the American Medical Association, 1881.

The Trance State in Inebriety: Its Medico-Legal Relations. By T. D. Crothers, M.D., with an introduction on the nature and character of the trance state by George M.

Beard, M.D. Read before the New York Medico-Legal Society.

Observations on the part the obstetrical forceps plays in the induction and prevention of perineal lacerations. By Thomas A. Ashby, M.D. Reprint from *Maryland Medical Journal.*

Questions submitted to the graduating classes of the Medical College of Ohio from 1871–72 to the present time. Cincinnati: Murray & Co., 1881.

